# Three-Week Versus 4-Week Schedule of *nab*-Paclitaxel in Patients With Metastatic Breast Cancer: A Randomized Phase II Study

**DOI:** 10.1093/oncolo/oyad288

**Published:** 2023-10-26

**Authors:** Yaxin Liu, Guohong Song, Lijun Di, Hanfang Jiang, Ran Ran, Ruyan Zhang, Yan Zhang, Huiping Li

**Affiliations:** Key Laboratory of Carcinogenesis and Translational Research (Ministry of Education/Beijing), Department of Breast Oncology, Peking University Cancer Hospital and Institute, Beijing, People’s Republic of China; Key Laboratory of Carcinogenesis and Translational Research (Ministry of Education/Beijing), Department of Breast Oncology, Peking University Cancer Hospital and Institute, Beijing, People’s Republic of China; Key Laboratory of Carcinogenesis and Translational Research (Ministry of Education/Beijing), Department of Breast Oncology, Peking University Cancer Hospital and Institute, Beijing, People’s Republic of China; Key Laboratory of Carcinogenesis and Translational Research (Ministry of Education/Beijing), Department of Breast Oncology, Peking University Cancer Hospital and Institute, Beijing, People’s Republic of China; Key Laboratory of Carcinogenesis and Translational Research (Ministry of Education/Beijing), Department of Breast Oncology, Peking University Cancer Hospital and Institute, Beijing, People’s Republic of China; Key Laboratory of Carcinogenesis and Translational Research (Ministry of Education/Beijing), Department of Breast Oncology, Peking University Cancer Hospital and Institute, Beijing, People’s Republic of China; Key Laboratory of Carcinogenesis and Translational Research (Ministry of Education/Beijing), Department of Breast Oncology, Peking University Cancer Hospital and Institute, Beijing, People’s Republic of China; Key Laboratory of Carcinogenesis and Translational Research (Ministry of Education/Beijing), Department of Breast Oncology, Peking University Cancer Hospital and Institute, Beijing, People’s Republic of China

**Keywords:** *nab*-paclitaxel, dose schedule, metastatic breast cancer, HER2-negative, prospective randomized control trial

## Abstract

**Background:**

This head-to-head study compared a 3-week versus 4-week schedule of *nab*-paclitaxel in patients with metastatic breast cancer (mBC).

**Methods:**

Patients with HER2-negative mBC were enrolled and randomly assigned (1:1) to receive *nab*-paclitaxel for a 3-week schedule (125 mg/m^2^ on days 1 and 8) or a 4-week schedule (same dose on days 1, 8, and 15) until disease progression or treatment intolerance. Patients with intolerable toxicities were allowed to receive a maintenance regimen after benefiting from *nab*-paclitaxel. The primary endpoint was progression-free survival (PFS).

**Results:**

Ninety-four patients were included in the analysis (*n* = 47 in each arm). A longer median PFS (mPFS) was observed in the 3-week versus the 4-week schedule in the overall population (not reached vs. 6.8 months; hazard ratio [HR] = 0.44; *P* = .029). Patients in the 2 arms had a similar overall survival (28.0 vs. 25.8 months), objective response rate (51.1% vs. 48.9%), and disease control rate (93.6% vs. 80.9%). The 3-week schedule was associated with a lower rate of toxicity-related treatment discontinuation (8.5% vs. 29.8%) and dose delays (6.4% vs. 23.4%).

**Conclusion:**

This study demonstrated the better antitumor activity and safety profile of a 3-week over 4-week *nab*-paclitaxel schedule in HER2-negative mBC, suggesting that a 3-week schedule may be a better treatment regimen in clinical practice (ClinicalTrials.gov Identifier: NCT04192331).

## Teaser

This study compared a 3-week versus 4-week schedule of *nab* -paclitaxel in patients with metastatic breast cancer.

**Lessons Learned**

- This study demonstrated the better safety profile and antitumor activity of a 3-week schedule of *nab*-paclitaxel compared with a 4-week schedule in patients with HER2-negative metastatic breast cancer.
- A 3-week schedule yielded an improved safety profile, better compliance, and fewer hospital visits.

## Discussion

Taxanes are considered among the most active chemotherapy agents in the standard treatment paradigm for mBC.^1^ However, conventional taxane-associated hypersensitivity reactions and neuropathy are significant challenges for clinicians and patients.^2^*nab*-paclitaxel is a novel solvent-free formulation developed to overcome some of the problems associated with solvent-based paclitaxel and has been recommended widely for mBC. However, a higher dose of *nab*-paclitaxel qw3/4 (150 mg/m^2^) is poorly tolerated, leading to dose reduction in at least 47% of patients.^3^ Therefore, optimizing dose schedules considering both favorable efficacy and tolerability profile remain an urgent need to improve patient compliance.

**Figure 1.**
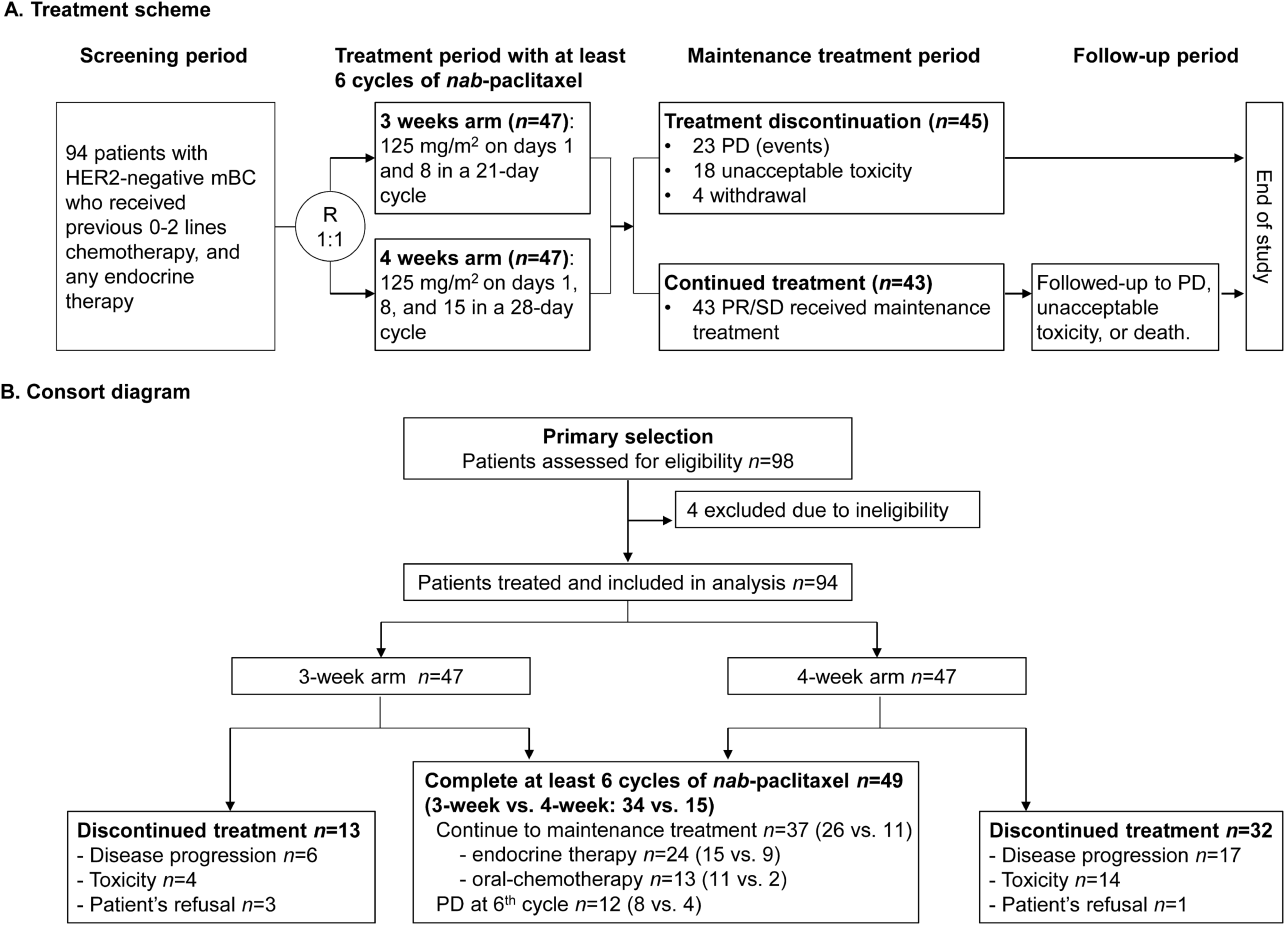
Treatment scheme (**A**) and CONSORT diagram (**B**).

Therefore, we performed a head-to-head randomized phase II study to compare a 3-week versus 4-week schedule of 125 mg/m^2^*nab*-paclitaxel. Between March 2019 and July 2021, among 98 patients assessed for eligibility, 94 patients were randomly assigned to either the 3-week arm (*n* = 47) or the 4-week arm (*n* = 47) (Fig. 1). At the cutoff date (December 26, 2022), after censoring patients who started maintenance therapy prior to progression because of toxicity, the median PFS (mPFS) was not reached in the 3-week arm because of insufficient events, compared with 6.8 months (95% CI [CI], 3.1-10.6) in the 4-week arm (HR = 0.44; 95% CI, 0.20-0.94; *P* = .029). However, similar results were observed for the 2 arms regarding the secondary endpoints (overall survival, 28.0 vs. 25.8 months; objective response rate, 51.1% vs. 48.9%; and disease control rate, 93.6% vs. 80.9%). In the subgroup analysis, a benefit of the 3-week regimen with respect to PFS was observed in the various prespecified subgroups, including patients with high Ki67 expression, HR-positive status, and visceral disease. More importantly, a more favorable safety profile was observed for the 3-week schedule, with fewer dose delays caused by adverse events (AEs; 6.4% vs. 23.4%), fewer ≥grade 3 AEs (14.9% vs. 42.6%), and other AEs of any grade (neutrophil count decrease, 59.6% vs. 78.7%; total bilirubin increase, 6.4% vs. 21.3%; and vomiting, 4.3% vs. 21.3%).

In summary, this study of HER2-negative mBC demonstrated a better efficacy and more favorable safety profile for the 3-week *nab*-paclitaxel schedule compared with the 4-week schedule.

**Table:** 

Trial Information	
Disease	HER2-negative metastatic breast cancer
Stage of disease/treatment	Stage IV/at 1-3 lines chemotherapy in metastatic setting
Prior therapy	No more than 2 prior lines of chemotherapy
Type of study	Randomized, phase II trial
Primary endpoint	Progression-free survival (PFS)
Secondary endpoints	Objective response rate (ORR), disease control rate (DCR), overall survival (OS), and safety
Investigator’s analysis	Active and should be pursued further

## Additional Details of Endpoints or Study Design

### Patient Inclusion Criteria

This was a single-center, open-label, prospective phase II randomized control trial that enrolled female patients aged ≥18 years who were histologically diagnosed with HER2-negative mBC at the Beijing Cancer Hospital and received no more than 2 prior lines of chemotherapy. Patients had to have measurable disease based on Response Evaluation Criteria in Solid Tumors (RECIST) criteria, or lytic or mixed bone lesions in the absence of measurable disease, as well as an Eastern Cooperative Oncology Group (ECOG) performance status of 0-1 and adequate organ and bone marrow function. Patients with uncontrolled brain metastases, congestive heart failure (NYHA class ≥II), previous peripheral nerve injury (≥grade II), allergy to study drugs or severe systemic infection were excluded.

The study protocol and patient materials were approved by the independent Ethics Committee of Peking University Cancer Hospital & Institute (Approval No. 2018YJZ52). All participants provided informed consent before enrollment. This study was registered with clinicaltrials.gov (Identifier, NCT04192331).

### Study Procedures

Eligible patients were randomly assigned in a 1:1 ratio to 2 treatment arms: a 3-week regimen (125 mg/m^2^*nab*-paclitaxel on days 1 and 8 in a 21-day cycle) or a 4-week regimen (125 mg/m^2^*nab*-paclitaxel on days 1, 8, and 15 in a 28-day cycle). *nab*-paclitaxel was administered intravenously, and the treatment cycle was decided by investigators according to the tumor response and tolerability. For patients with sustained tumor response and good tolerance, persistent treatment could be recommended. Patients with stable disease and poor tolerance were allowed to receive endocrine therapy or oral chemotherapy as maintenance treatment if they received over 6 cycles of chemotherapy. Because of the different administration schedules between the 2 arms, the patients and physicians were not blinded to treatment allocation. Dose reductions or delays were permitted in case of unacceptable toxicity. To avoid repeated hospital visits caused by AEs during the COVID-19 pandemic, most patients with poor bone marrow reserves were administered prophylactic colony stimulating factor (G-CSF) in the 2 arms.

### Endpoints and Assessment

The primary endpoint was progression-free survival (PFS), the time from randomization to disease progression or death from any cause, whichever occurred first. The secondary endpoints included the objective response rate (ORR, defined as the proportion of patients with the best overall response of the complete response [CR] and partial response [PR]) as per RECIST version 1.1, disease control rate (DCR, defined as the proportion of patients with CR, PR, and stable disease [SD]), overall survival (OS, defined as the time from randomization to death from any cause), and safety. Radiologic assessment was performed by 2 independent investigators every 8 weeks during the treatment period, until disease progression, death, or consent withdrawal. The investigator assessed AEs were assessed and classified according to severity grade using the National Cancer Institute Common Terminology Criteria for Adverse Events (NCI-CTCAE) version 5.0.

### Statistical Analysis

The trial was powered to assess PFS between the 2 different dose schedules of *nab*-paclitaxel. Assuming an equal median PFS of 5 months,^4,5^ a hazard ratio (HR) of 0.40 for the 3-week arm, an overall type I error of 2-sided 0.05, and a power of 80%, a total of approximately 37 progression events or deaths were required for the analysis. The study initially planned to randomly assign 94 patients (47 in each arm), assuming a 10% dropout rate.

Efficacy analyses were performed in the full analysis set (FAS) population who had received at least one dose of the study drug. For the primary analysis of PFS, data for patients who were alive and did not have disease progression, lost to follow up, or received non-study antitumor treatment before gaining evidence of objective tumor progression were censored to exclude the effects of maintenance therapy on PFS. To consider the impact of maintenance therapy in the sensitivity analysis of PFS, data from patients who were alive and did not have disease progression or who were lost to follow up were censored at the time of the last tumor assessment. For the analysis of OS, data from patients who were alive or lost to follow up were censored at the time of the last follow-up. The Kaplan-Meier method generated time-to-event curves, from which median values were calculated. A stratified log-rank test was used to compare the treatment groups, with an estimation of the HR and 95% CIs (CIs) from the log-rank test statistics. The relative dose intensity (RDI) was calculated as the actual dose intensity divided by the planned dose intensity during treatment. All statistical tests were 2-sided, with significance at *P* < .05.

**Table:** 

Drug Information: Multiarm		
	Arm 1: 3-week regimen	Arm 2: 4-week regimen
Generic/working name	*nab*-paclitaxel	*nab*-paclitaxel
Company name	CSPC Ouyi Pharmaceutical Co., Ltd.	CSPC Ouyi Pharmaceutical Co., Ltd.
Drug type	Cytotoxic agents	Cytotoxic agents
Drug class	Taxane	Taxane
Dose	125 mg/m^2^	125 mg/m^2^
Route	Intravenous (i.v.)	Intravenous (i.v.)
Schedule of administration	On days 1 and 8 in a 21-day cycle	On days 1, 8, and 15 in a 28-day cycle

**Table:** 

Patient Characteristics		
	Arm 1: 3-week arm	Arm 2: 4-week arm
Number of patients, male	0	0
Number of patients, female	47	47
Stage IV	47 (100%)	47 (100%)
Age, years, median (range)	57.0 (48.0-64.0)	50.0 (27.0-70.0)
≤65	39 (83.0%)	41 (87.2%)
>65	8 (17.0%)	6 (12.8%)
Performance status: ECOG	0: 39	0: 37
	1: 8	1: 10
	2: 0	2: 0
	3: 0	3: 0
	4: 0	4: 0
Number of metastatic sites	1: 5 (10.6%)	1: 3 (6.4%)
	2: 18 (38.3%)	2: 13 (27.7%)
	≥3: 24 (51.1%)	≥3: 31 (66.0%)
Number of prior systemic therapies: median (range)	1.0 (0.0-5.0)	1.0 (0.0-8.0)
Cancer types or histologic subtypes	HR+/HER2−: 38 (80.9%) Triple-negative breast cancer: 9 (19.1%)	HR+/HER2−: 40 (85.1%) Triple-negative breast cancer: 7 (14.9%)

**Table:** 

Primary Assessment Method: Antitumor Activity		
Arms	Arm 1: 3-week arm	Arm 2: 4-week arm
Number of patients screened	98	98
Number of patients enrolled	47	47
Number of patients evaluable for toxicity	47	47
Number of patients evaluated for efficacy	47	47
Evaluation method	RECIST 1.1	RECIST 1.1

**Table:** 

Response assessment	Arm 1: 3-week arm		Arm 2: 4-week arm	
	*N*	%	*N*	%
CR	0	0	0	0
PR	24	51.1	23	48.9
SD	20	42.6	15	31.9
PD	3	6.4	9	19.1
ORR	24	51.1 (95% CI, 36.1-65.9)	23	48.9 (95% CI, 34.1-63.9)
DCR	44	93.6 (95% CI, 82.5-98.7)	38	80.9 (95% CI, 66.7-90.9)

**Table:** 

(Median) Duration assessments	Arm 1: 3-week arm	Arm 2: 4-week arm
	Months	Months
PFS*	Not reached	6.8 (95% CI, 3.1-10.6)
OS	28.0 (95% CI, 21.4-34.6)	25.8 (95% CI, 17.3-34.3)
Duration of treatment	3.9 (IQR, 2.9-5.4)	3.5 (IQR, 2.3-5.2)

## Outcome Notes

**Figure 2.**
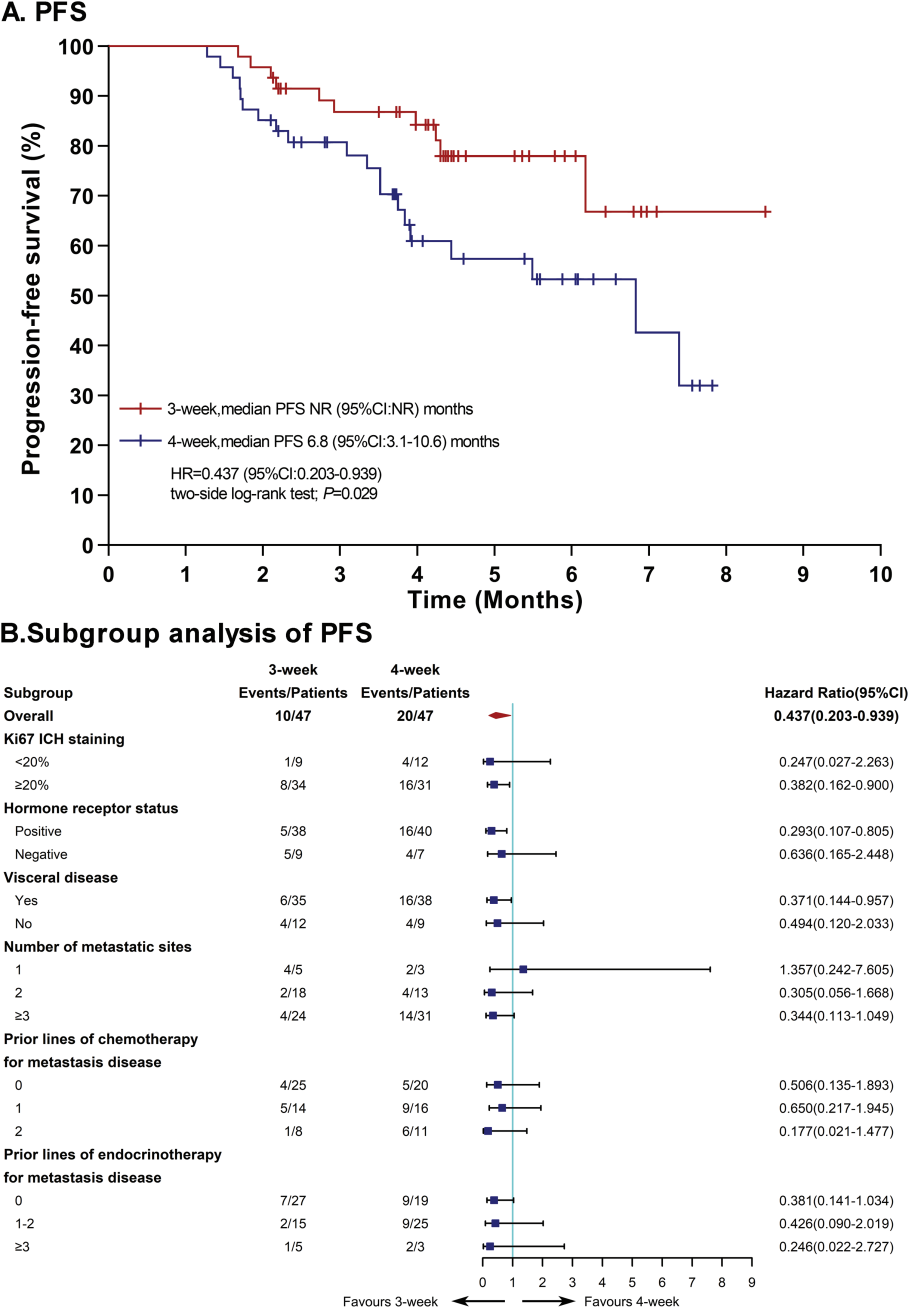
Efficacy outcomes of progression-free survival (PFS) in the FAS population. (**A**) After censoring patients who started maintenance therapy prior to progression because of toxicity, Kaplan-Meier survival curves of PFS demonstrating a better PFS in the 3-week arm versus the 4-week arm (not reached versus 6.8 months; HR = 0.44, 95% CI, 0.20-0.94; *P* = .029); PFS was assessed by the investigators according to RECIST version 1.1. (**B**) Analysis of PFS in key subgroups based on baseline characteristics. *PFS was analyzed in the FAS population and censored patients who were alive, had no disease progression, were lost to follow up, or received subsequent maintenance therapy before progression to exclude the effects of maintenance therapy on PFS.

**Figure 3.**
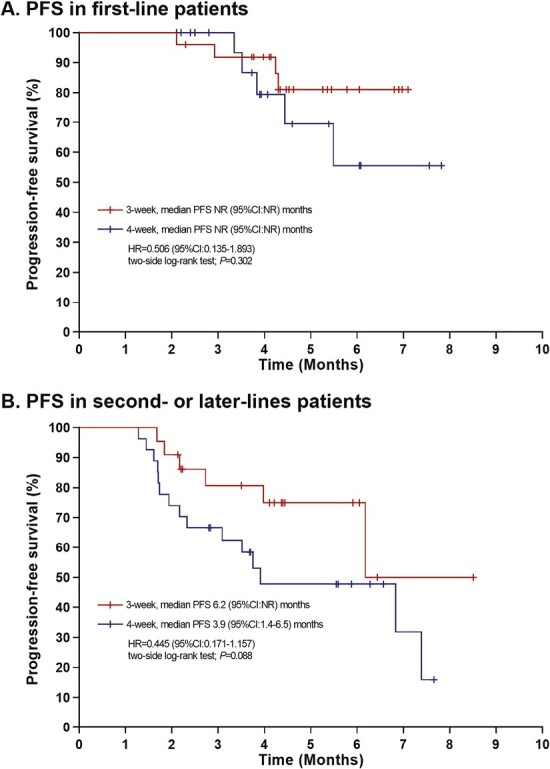
Efficacy outcomes of progression-free survival (PFS) as classified by the patients. (**A**) Kaplan-Meier survival curves of PFS demonstrating a similar result in first-line patients; (**B**) Kaplan-Meier survival curves of PFS demonstrating a better tendency in the 3-week arm versus the 4-week arm in second- or later-line patients (6.2 vs. 3.9 months).

**Figure 4.**
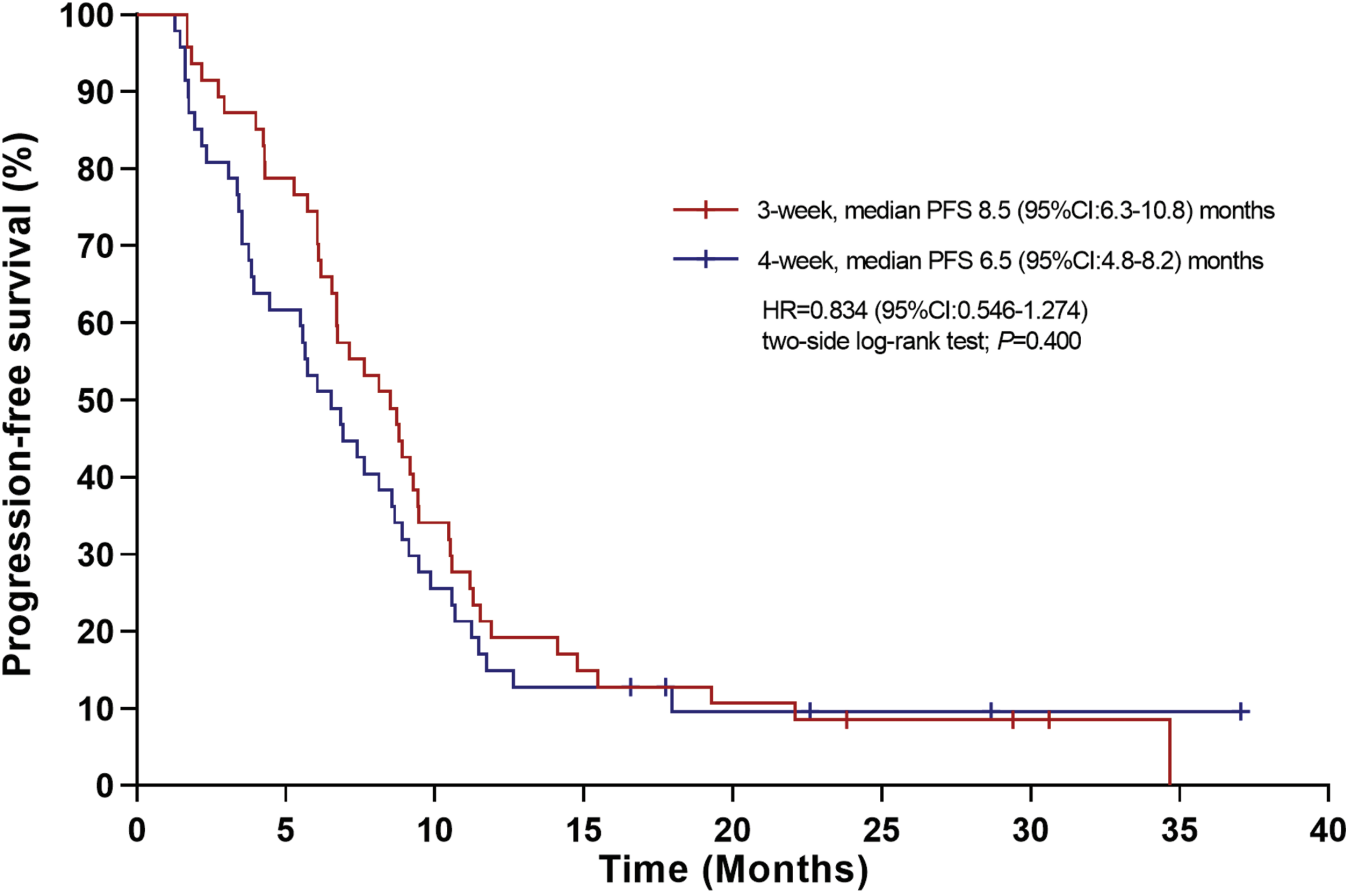
Kaplan-Meier survival curves of PFS in the sensitivity analysis. During the whole treatment period, the 3-week schedule of *nab*-paclitaxel yielded a better tendency of PFS compared with the 4-week schedule (8.5 [95% CI, 6.3-10.9] versus 6.5 [95% CI, 4.8-8.2] months), with an HR of 0.83 (95% CI, 0.55-1.274; *P* = .400).

**Figure 5.**
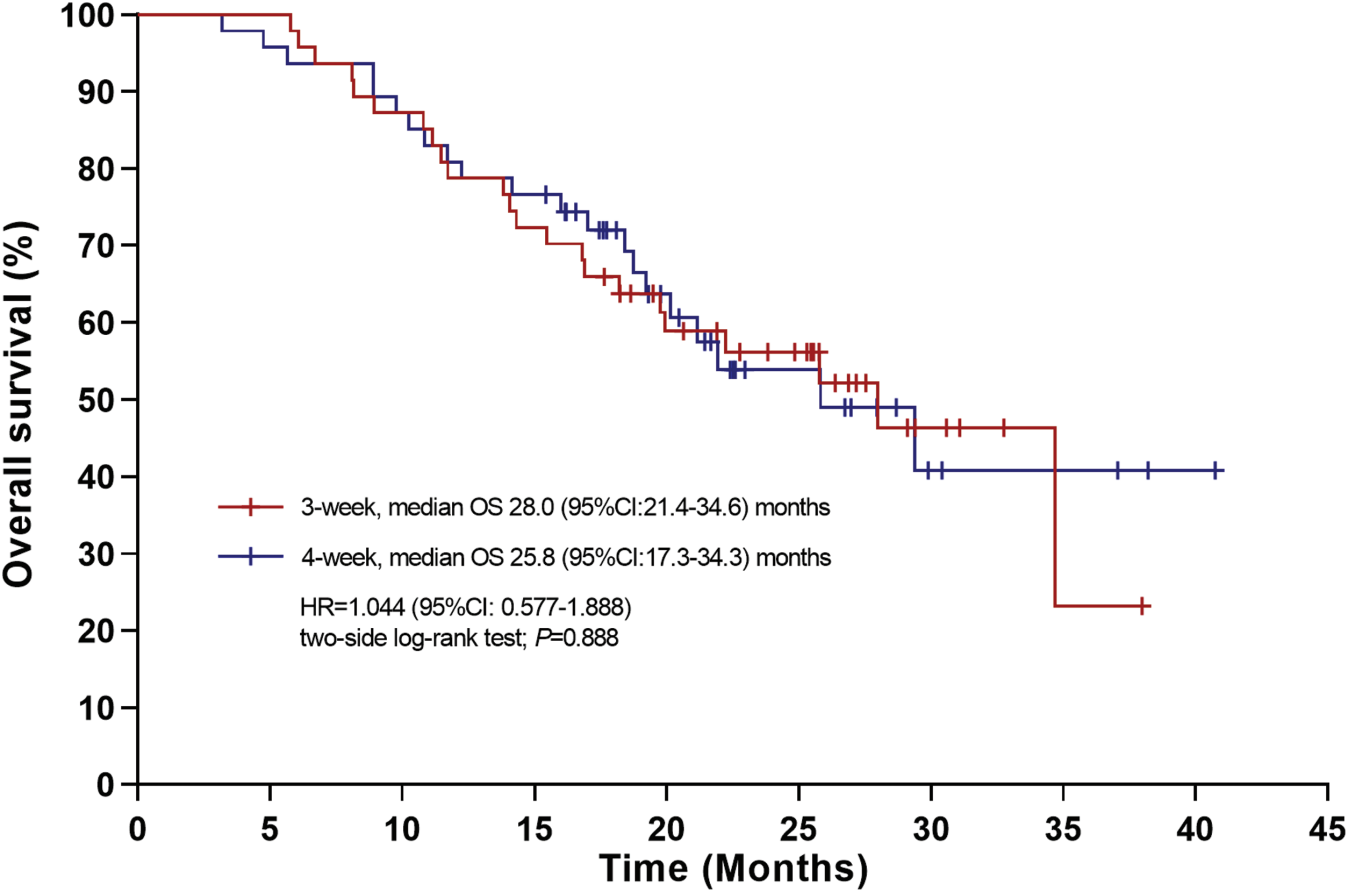
Kaplan-Meier survival curves of overall survival (OS). The median OS was 28.0 (95% CI, 21.4-34.6) months in the 3-week arm and 25.8 (95% CI, 17.3-34.3) months in the 4-week arm. There was no significant OS difference between the 2 arms (HR = 1.04; 95% CI, 0.58-1.89; *P* = .888).

See Figs. 1-5, Table 1 (baseline demographic and clinical characteristics), and Table 2 (treatment exposure and dose modifications). All 94 patients in the safety analysis set reported at least one emergent AE (Table 3).

**Table 1.** Baseline demographic and clinical characteristics.

	3-week arm (*n* = 47)	4-week arm (*n* = 47)	*P*-value
Age			.562
≤65 years	39 (83.0)	41 (87.2)	
>65 years	8 (17.0)	6 (12.8)	
Subtype			.583
HR+/HER2−	38 (80.9)	40 (85.1)	
TNBC	9 (19.1)	7 (4.9)	
Visceral disease^*^	35 (74.5)	38 (80.9)	.458
Metastatic site			—
Liver	22 (46.8)	27 (57.4)	
Lung	21 (44.7)	23 (48.9)	
Bone	23 (48.9)	25 (53.2)	
Brain	2 (4.3)	1 (2.1)	
Thoracic wall	9 (19.1)	7 (14.9)	
Serous effusion	4 (8.5)	4 (8.5)	
Number of metastatic sites			.398
1	5 (10.6)	3 (6.4)	
2	18 (38.3)	13 (27.7)	
≥3	24 (51.1)	31 (66.0)	
Treatment-naïve stage IV	9 (19.1)	9 (19.1)	>.999
DFS, months median (range)	45.7 (0.0, 166.3)	35.0 (0.0, 177.7)	.614
Prior lines of chemotherapy for metastasis disease			.547
0	25 (53.2)	25 (53.2)	
1	14 (29.8)	14 (29.8)	
2	8 (17.0)	8 (17.0)	
Prior lines of endocrinotherapy for metastasis disease			.125
0	27 (57.4)	19 (40.4)	
1-2	15 (31.9)	25 (53.2)	
≥3	5 (10.6)	3 (6.4)	

Data are median (range) or *n* (%).

^*^Visceral disease included patients with lung, liver, brain, or peritoneal metastases.

Abbreviation: DFS: disease-free survival.

**Table 2.** Treatment exposure and dose modifications.

	3-week arm (*n* = 47)	4-week arm (*n* = 47)	*P-*values
*Number of treatment cycles*			<.001
Total cycles, *n*	276	203	
Mean ± SD	5.9 ± 2.0	4.3 ± 1.8	
Median (range)	6.0 (2.0, 10.0)	4.0 (2.0, 8.0)	
*Treatment cycles, n (%)*			<.001
<6	13 (27.7)	32 (68.1)	
≥6	34 (72.3)	15 (31.9)	.224
First-line therapy	20 (58.8)	6 (40.0)	
Second/third-line therapy	14 (41.2)	9 (60.0)	
*Relative dose intensity* ^*^ *, %*			.345
Mean ± SD	94.6 ± 8.4	92.9 ± 8.4	
Median (range)	94.2 (80.6, 114.3)	93.8 (71.7, 109.2)	
*Relative dose intensity (binary classification), n (%)*			.241
≤80%	0 (0.0)	3 (6.4)	
>80%	47 (100.0)	44 (93.6)	
*Patients with ≥1 cycle delays, n (%)*	7 (14.9)	15 (31.9)	.051
Due to AEs, *n* (%)	3 (6.4)	11 (23.4)	.020
*Patients with ≥1 dose reduction, n (%)*	1 (2.1)	2 (4.3)	>.999
Due to AEs, *n* (%)	1 (2.1)	2 (4.3)	>.999
*Treatment discontinuation due to AEs, n (%)*	4 (8.5)	14 (29.8)	.009
*Maintenance therapy, n (%)*	26 (55.3)	11 (23.4)	.002
Endocrine	15 (57.7)	9 (81.8)	
Oral-chemotherapy	11 (42.3)	2 (18.2)	

Data are mean (SD) or *n* (%).

^*^Relative dose intensity was calculated as the actual dose intensity divided by the planned dose intensity during the entire treatment duration.

**Table 3.** Summary of adverse events.

AEs, *n* (%)	3-week arm (*n* = 47)		4-week arm (*n* = 47)	
	Any grade	≥3 grade	Any grade	≥3 grade
Hematological				
White blood cell decreased	35 (74.5)	5 (10.6)	39 (83.0)	9 (19.1)
Neutrophil count decreased	28 (59.6)	6 (12.8)	37 (78.7)	16 (34.0)
Anemia	26 (55.3)	1 (2.1)	26 (55.3)	1 (2.1)
Platelet count decreased	3 (6.4)	0 (0.0)	1 (2.1)	0 (0.0)
Non-hematological (>5% incidence)				
Peripheral neuropathy	35 (74.5)	0 (0.0)	36 (76.6)	2 (4.3)
Electrocardiogram abnormal	34 (72.3)	0 (0.0)	33 (70.2)	0 (0.0)
Malaise	31 (66.0)	0 (0.0)	37 (78.7)	0 (0.0)
Alopecia	31 (66.0)	0 (0.0)	33 (70.2)	0 (0.0)
Ala9 aminotransferase increased	25 (53.2)	0 (0.0)	20 (42.6)	0 (0.0)
Aspartate aminotransferase increased	21 (44.7)	0 (0.0)	19 (40.4)	0 (0.0)
Rash	17 (36.2)	0 (0.0)	19 (40.4)	1 (2.1)
Nausea	17 (36.2)	0 (0.0)	19 (40.4)	0 (0.0)
Arthralgia	14 (29.8)	0 (0.0)	15 (31.9)	1 (2.1)
Hand-foot syndrome	7 (14.9)	0 (0.0)	11 (23.4)	1 (2.1)
Diarrhea	6 (12.8)	0 (0.0)	10 (21.3)	0 (0.0)
Direct bilirubin increased	4 (8.5)	0 (0.0)	8 (17.0)	1 (2.1)
Constipation	4 (8.5)	0 (0.0)	7 (14.9)	0 (0.0)
Total bilirubin increased	3 (6.4)	0 (0.0)	10 (21.3)	0 (0.0)
Edema limbs	3 (6.4)	0 (0.0)	7 (14.9)	0 (0.0)
Fever	3 (6.4)	0 (0.0)	2 (4.3)	0 (0.0)
Vomiting	2 (4.3)	0 (0.0)	10 (21.3)	0 (0.0)
Indirect bilirubin increased	2 (4.3)	0 (0.0)	6 (12.8)	0 (0.0)

**Table:** 

Assessment, Analysis, and Discussion	
Completion	Study completed
Investigator’s assessment	Active and should be pursued further

Schedule modification is a common strategy in clinical oncology to optimize the therapeutic index of established cytotoxic agents. The present head-to-head randomized phase II study in HER2-negative mBC demonstrated a better efficacy and more favorable safety profile for the 3-week *nab*-paclitaxel schedule compared with the 4-week schedule, which should be recommended by guidelines in the future.

Several clinical trials have investigated alternative *nab*-paclitaxel doses and dosing schedules.^6-8^ Here, we used a dose of 125 mg/m^2^ qw2/3 because of its tolerability. This reduced dose is now widely accepted by many cancer centers in China^9^ and other Asian countries.^10,11^ This study was the first prospective study to compare the 3-week versus 4-week dose of 125 mg/m^2^. In this randomized trial, we observed a PFS benefit for the 3-week schedule over the 4-week schedule in HER2-negative mBC after excluding the impact of maintenance therapy. Despite the fact that mPFS was not reached in the 3-week arm, the early mPFS benefit detected after the 6-cycle *nab*-paclitaxel treatment was maintained in the 3-week versus the 4-week schedule, as evidenced by the significant separation of the Kaplan-Meier curve at 6 months and the 24.7% increase in the 6-month PFS rate (78.0% vs. 53.3%). Although maintenance therapy contributed to additional events, the PFS benefit observed during the *nab*-paclitaxel therapy afforded by the 3-week regimen seemed to have been maintained through to the end of the study because the 3-week regimen afforded a numerical benefit in PFS with a 2-month extension compared with the 4-week regimen (8.5 vs. 6.5 months), although this difference was not significant. The observed survival benefit associated with the 3-week schedule may be attributed to better patient compliance, ie, the patients completed more cycles of treatment in the 3-week arm (the ≥6-cycle completion rate was 72.3% in this arm, which was far better than the 31.9% observed in the 4-week arm); as well as to the lower toxicity-related treatment discontinuation rate and reduced dose delays recorded in the 3-week arm. These results demonstrated that a more tolerable treatment schedule might allow for a favorable balance between efficacy and quality of life in patients with metastatic breast cancer. The greater probability of a better outcome in the 3-week schedule may be attributed to the fact that a 21-day interval of dose delivery is the best response window for tumor cells, because it matches the tumor cell repair cycle, thus achieving a better therapeutic effect.^12^ Moreover, this explains why a 3-week schedule is mostly considered the standard regimen for most chemotherapies. In turn, data on the efficacy of the qw2/3 regimen used in this study seems better than previously reported data from various q3w dose regimens, with a PFS ranging from 6.66 to 7.34 months.^13^

There was no OS benefit in the 3-week regimen compared with the 4-week arm, which might be attributed to the complex situation of the treatment of mBC. In addition, patient compliance, endocrine sensitivity, and subsequent maintenance treatments may affect OS to a large extent. Consistently, the ORR (51.1% vs. 48.9%) and DCR (93.6% vs. 80.9%) also exhibited no notable differences between the 2 arms. Our results were generally comparable with the data from a previous trial reported by Tamura et al,^14^ in which *nab*-paclitaxel (as the first-line chemotherapy) achieved an ORR of 56.1% and a DCR of 92.9% in Asian patients with HER2-negative mBC.

The 3-week schedule was investigated in the hope of providing a better safety profile to the patients. Consistently, here, the 3-week schedule of *nab*-paclitaxel afforded a generally more favorable safety profile. Several AEs, including vomiting, blood bilirubin increase, and ≥grade 3 neutrophil count decrease, occurred more frequently in the 4-week arm. Notably, few dose reductions associated with *nab*-paclitaxel (2.1%-4.3%) occurred in the 2 arms, which was a better outcome than the data obtained from the 4-week schedule of 100-150 mg/m^2^*nab*-paclitaxel (~50%-70%).^3,7^ The prophylaxis for neutropenia administered during the COVID-19 pandemic might explain the fewer events of dose reduction observed in our study. The common AEs were similar to those of previous studies.^8,15^ Overall, with the goal of obtaining a favorable safety profile and better compliance combined with fewer hospital visits, the 3-week schedule is recommended with priority.

Several limitations of the present study should be recognized. First, this analysis was based on a small sample, which may have led to a hidden bias. Second, a small proportion of patients in the 2 arms had delayed treatment because of the COVID-19 pandemic, which might have affected the interpretation of the efficacy results. However, the calculated RDI data showed that the patients in 2 arms comparability received more than 80% of the planned dose intensities because of the prophylactic use of G-CSF, and this RDI level also ensured the anticipated efficacy well.^16,17^ Further confirmation of a better PFS and a comparison of long-term benefits remain necessary.

In summary, this study demonstrated the better safety profile and anti-cancer activity of the 3-week schedule of *nab*-paclitaxel compared with the 4-week schedule in patients with HER2-negative mBC. Because of the superiority in the patient adherence to the 3-week schedule, this might be a better regimen in clinical practice. The optimal *nab*-paclitaxel dose and dosing schedule remain an open question and should be confirmed in future studies.

## Data Availability

The data underlying this article will be shared on reasonable request to the corresponding author.
